# Clozapine delirium treated with anesthesia: a case report

**DOI:** 10.1192/j.eurpsy.2026.11330

**Published:** 2026-07-31

**Authors:** T. Mastelić, M. Ivandić, T. Vukićević, D. Lasić

**Affiliations:** Department of Psychiatry, University Hospital of Split, Split, Croatia

## Abstract

**Introduction:**

Clozapine is an antipsychotic commonly used in treatment-resistant schizophrenia. Although it is very effective, it has numerous side effects. In addition to the problems of leukopenia and agranulocytosis, delirium is a particular side effect. Clozapine-induced delirium usually occurs when the dose of clozapine is increased, at higher doses, or with polypharmacy. It is assumed that infections or inflammation can lead to higher levels of clozapine in the blood. The mechanism of delirium is not completely clear, but it is believed to be a consequence of the anticholinergic effects of clozapine and a cholinergic imbalance. Delirium is characterized by confusion, disorientation, agitation, and memory problems.

**Objectives:**

To present the case of a person who is being treated for schizophrenia and was intoxicated with clozapine, which led to the development of strong delirium that subsided only after the application of general anesthesia.

**Methods:**

Review of medical records.

**Results:**

A patient in his 20s, who was being treated for schizophrenia and had been hospitalized several times, became intoxicated with clozapine and olanzapine. He took approximately 480 mg of olanzapine and 1050 mg of clozapine with self-destructive intent. He was found sedated and taken to the emergency department. Shortly after admission to the intensive care unit, the patient developed severe restlessness, to the point of agitation, with disorientation and confusion. 40 mg of diazepam, 10 mg of haloperidol, and 30 mg of midazolam were used to calm the patient. None of the above helped, and the patient finally calmed down only after he was anesthetized, intubated, and placed on mechanical ventilation. The patient later had no memory of the events described above.

**Conclusions:**

In the presented case, we can see an example of a patient who developed severe delirium due to intoxication with large amounts of clozapine, in whom conventional treatment methods did not yield results. We believe that it is worth recording such a case, in which sedation had to be performed under general anesthesia.

**Disclosure of Interest:**

None Declared

